# Evidence for isotropic s-wave superconductivity in high-entropy alloys

**DOI:** 10.1038/s41598-022-16355-4

**Published:** 2022-07-27

**Authors:** Casey K. W. Leung, Xiaofu Zhang, Fabian von Rohr, Rolf Lortz, Berthold Jäck

**Affiliations:** 1 Department of Physics, The Hong Kong University of Science and Technology, Clear Water Bay, Kowloon, Hong Kong SAR China; 2 State Key Laboratory of Functional Materials for Informatics, Shanghai Institute of Microsystem and Information Technology, Chinese Academy of Sciences (CAS), Shanghai, 200050 China; 3 CAS Center for Excellence in Superconducting Electronics, Shanghai, 200050 China; 4 Department of Chemistry, Universiät Zürich, Winterthurerstrasse 190, 8057 Zurich, Switzerland; 5 IAS Center for Quantum Technologies, The Hong Kong University of Science and Technology, Clear Water Bay, Kowloon, Hong Kong SAR China

**Keywords:** Condensed-matter physics, Superconducting properties and materials

## Abstract

High-entropy alloys (HEA) form through the random arrangement of five or more chemical elements on a crystalline lattice. Despite the significant amount of resulting compositional disorder, a subset of HEAs enters a superconducting state below critical temperatures, $T_{\text{c}}<10\,$ K. The superconducting properties of the known HEAs seem to suffice a Bardeen–Cooper–Schrieffer (BCS) description, but little is known about their superconducting order parameter and the microscopic role of disorder. We report on magnetic susceptibility measurements on films of the superconducting HEA (TaNb)$_{1-x}$(ZrHfTi)$_{x}$ for characterizing the lower and upper critical fields $H_{\text{c,1}}(T)$ and $H_{\text{c,2}}(T)$, respectively as a function of temperature *T*. Our resulting analysis of the Ginzburg–Landau coherence length and penetration depth demonstrates that HEAs of this type are single-band isotropic s-wave superconductors in the dirty limit. Despite a significant difference in the elemental composition between the $x=0.35$ and $x=0.71$ films, we find that the observed $T_{\text{c}}$ variations cannot be explained by disorder effects.

## Introduction

High-entropy alloys (HEAs) are a new type of alloy with five or more chemical elements arranged on a pseudocrystalline lattice^1–6^. A high mixing-entropy minimizes the Gibbs free-energy and facilitates their crystallization on simple lattice structures, such as body-centered cubic (bcc) structure^1,2,4,5^. Despite the significant amount of compositional disorder, a subset of the HEAs enters a type-II superconducting phase at cryogenic temperatures^7,8^. Their large critical fields combined with superior mechanical and thermal properties^8–12^ render HEA promising candidates for materials applications under extreme conditions, for the fabrication of superconducting magnets, and for superconducting devices based on HEA thin films^13^.

Ongoing materials synthesis efforts have extended the family of known superconducting HEAs and developed a phenomenological understanding of their properties^8,14–19^. Various analyses of the upper critical field $H_{\text{c2}}$ and heat capacity measurements as a function of temperature *T* support a conventional Bardeen–Cooper–Schrieffer (BCS) pairing mechanism^7,8,13,20^ with intermediate-strong coupling^20^. A dependence of the superconducting transition temperature $T_{\text{c}}$ on the chemical composition, as measured in the number of available valence electrons, and mixing entropy has been established^8,13,20^. Nevertheless, conclusive experimental insight on the superconducting order parameter, such as obtained from temperature-dependent penetration-depth measurements, and the influence of disorder on the superconducting state is missing to date.

**Figure 1:**
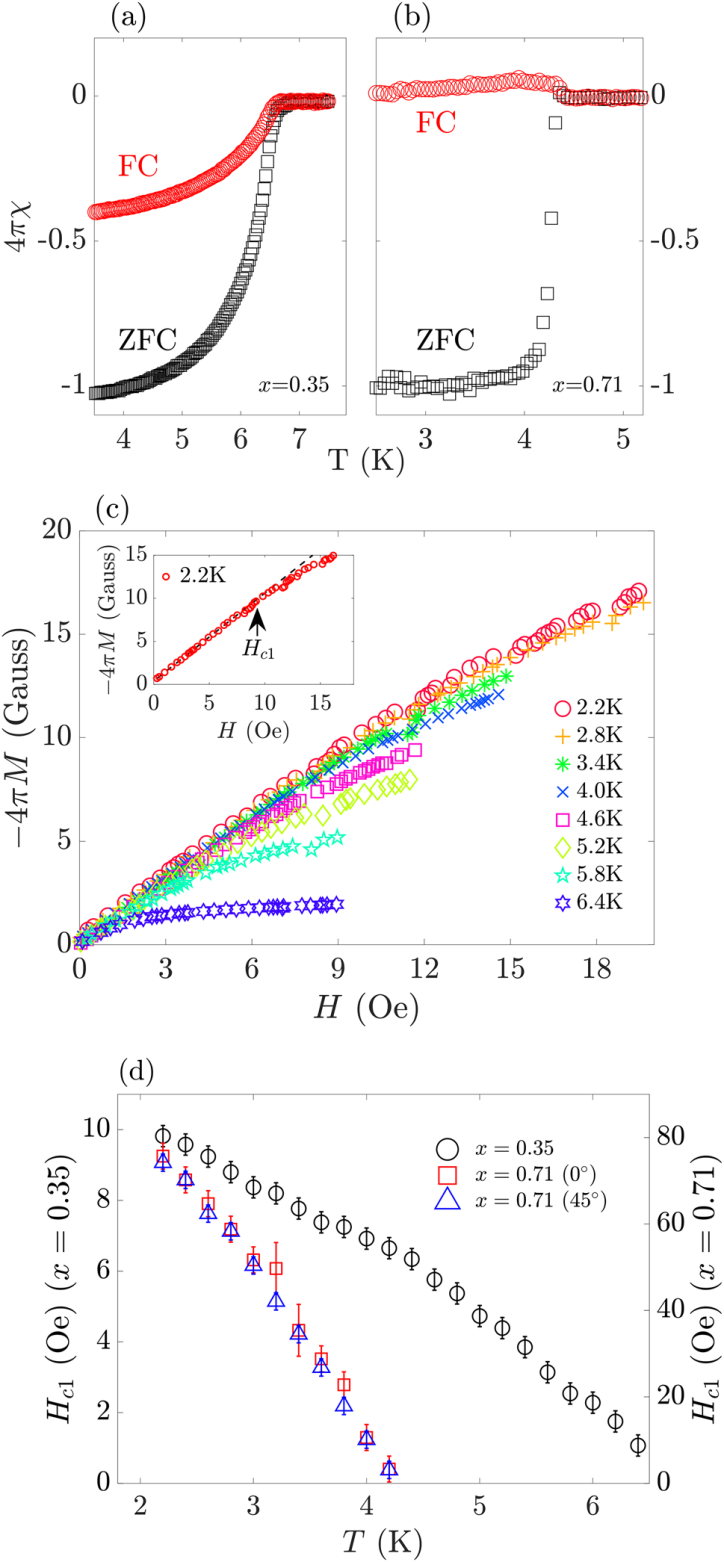
Zero-field cooling (ZFC) and field-cooling (FC) curves of the magnetic susceptibility $\chi$ as a function of temperature, *T*, for the HEA films with $x=0.35$ (**a**), and $x=0.71$ (**b**). ZFC (FC) measurements were conducted with $H=5\,$Oe ($H=50\,$ Oe) applied in parallel to the film plane. (**c**) Superconducting volume fraction $-\,4\pi M$ measured as a function of an external magnetic field *H* applied in parallel to the $x=0.35$ film at different temperatures indicated in the legend. The inset shows a linear fit to $-\,4\pi M(H)$ at $T=2.2\,$ K for determining the lower critical field $H_{\text{c1}}$. The corresponding experimental data for the $x=0.71$ film and details of the procedure for determining $H_{\text{c1}}$ are presented in Sect. B of the Supplementary Materials. (**d**) Fitted $H_{\text{c1}}(T)$ of the $x=0.35$ (left axis) and $x=0.71$ (right axis) films. Measurements with the magnetic field applied along two different in-plane angles, $0^{\circ }$ and $45^{\circ }$, are shown for the $x=0.71$ data.

It has long been recognized that a critical amount of disorder can suppress superconductivity near the Anderson quantum phase transition^21^. The observed $T_{\text{c}}$ reduction concomitant with a mixing entropy $\Delta S$ enhancement in superconducting HEAs seems to agree with that picture^20^. On the other hand, recent analyses of competing interaction-channels in strongly disordered metals predict a $T_{\text{c}}$ enhancement within the BCS framework, when the electron system is tuned to a quantum critical point^22,23^.

This hypothesis finds support in a recent study of HEA $({\text{TaNb}})_{{1-\text{x}}}({\text{HfZrTi}})_\text{{x}}$ thin films; while each of the binary alloys ${\text{TaNb}}$ and ${\text{HfZrTi}}$ does not show a superconducting transition at or above 2 K, all solid solutions of $(\text{{TaNb}})_{{1-\text{x}}}(\text{{HfZrTi}})_\text{{x}}$ at various mixing ratios *x* are superconducting at $T_\text{{c}}\le 6.9$ K^13^. This ’cocktail’ effect suggests an intricate relation between the presence of strong compositional disorder, realized through the random arrangement of five atomic species on a bcc lattice, and superconductivity, which is an interesting question to address experimentally.

In this letter, we report on temperature-dependent magnetization measurements of $(\text {TaNb})_{1-\text{x}}(\text {HfZrTi})_\text{x}$ HEAs at different alloy compositions *x* to characterize the superconducting state in more detail. Our analysis of the superconducting penetration-depth $\lambda (T)$ is in quantitative agreement with BCS theory for an isotropic single-band s-wave superconductor in the weak (to intermediate) coupling limit. Our experimental results further show that, despite the large amount of atomic scale disorder, the observed $T_\text{c}$ variations for films of different elemental compositions cannot be explained by disorder effects.

## Experiment

Films of superconducting (TaNb)$_{1-x}$(ZrHfTi)$_{x}$ with nominal $x=0.40$ and $x=0.75$ have been prepared by magnetron sputtering on the surface of SiN wafers as described in Ref.^13^. In contrast to other HEA types^24^, the zero or small binary mixing enthalpies of the constituent elements in TaNbZrHfTi HEAs favor the formation of a single phase structure, when depositing the HEA film on a substrate held at room temperature^13^. Consistent with our previous study of this compound^13^, the formation of a completely mixed single phase and the absence of other binary phases is confirmed by chemical mapping through energy-dispersive X-ray spectroscopy measurements with the scanning electron microscope. These measurements further facilitate the determination of the actual film compositions $x=0.35$ and $x=0.71$ that closely match the targeted compositions. X-ray diffraction measurements confirm the single-phase crystallization on a body-centered cubic lattice with a film thickness $d\approx 1\,{\upmu }$m. For details on the binary enthalpies, and the chemical and structural characterization please refer to Sect. A of the Supplementary Materials.

**Figure 2:**
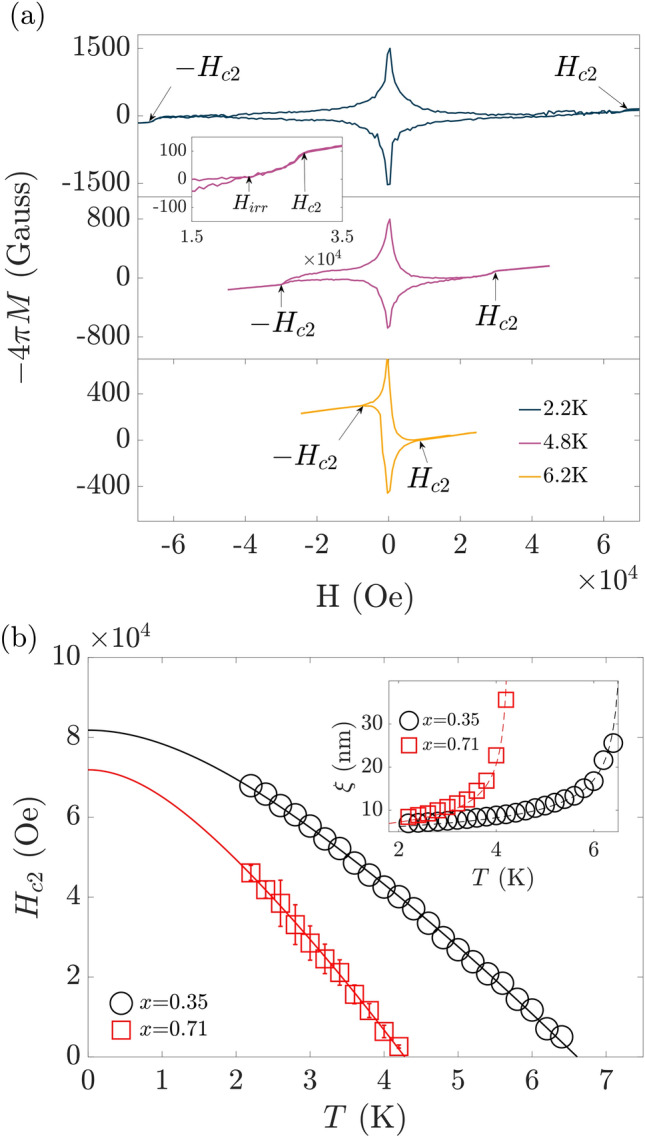
(**a**) Measurements of the superconducting volume fraction $-\,4\pi M$ as a function of the external magnetic field *H* applied perpendicular to the sample plane for the $x=0.35$ film. Shown are three representative measurements at indicated temperature, *T*. (**b**) Extracted temperature dependence of the upper critical field $H_\text{c2}(T)$ of the $x=0.35$ and $x=0.71$ films. The solid lines show the fits of $H_\text{c2}(T)$ with the Werthamer–Helfland–Hohenberg model^25^. The inset shows the corresponding temperature dependence of the Ginzburg–Landau coherence length $\xi$ of the $x=0.35$ and $x=0.71$ films. The dashed lines are the corresponding fits to $\xi (T)$ for superconductors in the dirty limit.

The molar mixing entropy $\Delta S=R\sum x_\text{i}\log (x_\text{i})$ (*R*—ideal gas constant), of the $x=0.71$ alloy, $\Delta S_\text{x=0.71} = -\,1.56\,R$, is comparable to that of the $x=0.35$ alloy, $\Delta S_\text{x=0.35} = -\,1.53\,R$. It is interesting to note that the (TaNb)$_{1-x}$(ZrHfTi)$_{x}$ alloys with similar mixing ratios $x=0.40$ and $x=0.75$ were reported to show the highest $T_\text{c}\le 7$ K and highest $H_\text{c}\approx 10\,$T, respectively^13^.We have performed high-resolution DC magnetization measurements using a commercial Quantum Design MPMS3 VSM-SQUID magnetometer under cryogenic conditions $T\ge 1.8$ K to characterize the superconducting state of the HEA samples. Measuring their magnetic susceptibility $\chi (T)$, we have determined their $T_\text{c}$, the lower $H_\text{c1}$ and upper $H_\text{c2}$ critical fields as a function of temperature and externally applied magnetic field *H*.

## Results

Zero-field cooling/Field cooling (ZFC/FC) measurement were performed to establish superconductivity in the HEA films, see Fig. 1a,b. Both the $x=0.35$ and $x=0.71$ film show a diamagnetic response with unity superconducting volume fraction in ZFC measurements at $T\ll T_\text{C}$. The extracted $T_\text{c}=(6.7\pm 0.1)\,$K and $T_\text{c}=(4.3\pm 0.1)\,$K of the $x=0.35$ and $x=0.71$ film, respectively are in agreement with previous reports^26^. The FC measurements further indicate strong flux pinning. The diamagnetic response of the $x=0.35$ film is suppressed by about 60%, whereas the $x=0.71$ film exhibits a small paramagnetic Meissner effect^27^.

The penetration depth of a superconductor can be determined through measurements of $H_\text{c1}$ and $H_\text{c2}$. We have determined $H_\text{c1}(T)$ by mapping out the field response of the HEA films at small external magnetic fields applied in parallel to the film plane. In Fig. 1c, we plot the corresponding superconducting volume fraction $-\,4\pi M(H)$ at different experimental temperatures for the $x=0.35$ film (see Sects. B,C of the Supplementary Materials for the corresponding data of the $x=0.71$ film and the superconducting volume fraction determination, respectively). At small applied fields, $-\,4\pi M(H)$ exhibits a linear dependence with a slope of unity. This observation is consistent with the diamagnetic response of bulk superconductivity in the HEA films.

The deviation from linearity at larger *H* occurs at $H_\text{c1}$ at which the HEA films enter the mixed phase, i.e., magnetic vortices are penetrating the superconducting volume. We have determined $H_\text{c1}$ as the field at which the measured $-\,4\pi M(H)$ data deviate from a linear fit to the small-field region, see Fig. 1c inset. The fitting procedure is described in Sect. D of the Supplementary Materials. The resulting $H_\text{c1}(T)$ is displayed in Fig. 1d. While $H_\text{c1}$ is strongly suppressed for both alloy compositions at $T\rightarrow T_\text{c}$, $H_\text{c1}$ of the $x=0.71$ film is about an order of magnitude larger compared to the $x=0.35$ film at $T\ll T_\text{c}$. Furthermore, other measurements show that $H_\text{c1}(T)$ is not affected by a 45% rotation of H in the sample plane (see Fig. 1d).

We have measured the magnetic susceptibility over a larger field range of $-\,70\,$ kOe $<H<+\,70\,$ kOe to further determine the temperature dependence of $H_\text{c2}$. In Fig. 2a, we plot the corresponding $-\,4\pi M(H)$ for representative measurements of the $x=0.35$ film (see Sect. D of the Supplementary Materials for the corresponding data of the $x=0.71$ film). We observe a significant magnetic hysteresis between forward and backward sweep, indicative of vortex pinning below the irreversibility field $H_\text{irr}$ (see inset of Fig. 2a). $H_\text{c2}$ can be determined from these measurements as the field, at which forward and backward trace deviate from the linear background signal, see marker in Fig. 2a. The resulting $H_\text{c2}(T)$ dependence is shown in Fig. 2b. We observe a monotonic, almost linear, decay of $H_\text{c2}(T)$ near $T_\text{c}$ for both alloys.

## Discussion

We can accurately describe $H_\text{c2}(T)$ by using the Werthamer–Helfland–Hohenberg (WHH) model of conventional superconductors in the presence of spin-paramagnetism and spin-orbit interaction (see Fig. 2b)^25^. Fitting $H_\text{c2}(T)$, we obtain $H_\text{c2, 0}=(81.8 \pm 0.4)\,$ kOe and $H_\text{c2, 0}=(71.9 \pm 0.6)\,$ kOe for the $x=0.35$ and $x=0.71$ film, respectively. These values are significantly smaller than the values of the corresponding Pauli paramagnetic limit in the weak coupling limit $H_\text{P}=18.4T_\text{C}$ ($H_\text{P}$ in kOe and $T_\text{C}$ in K)^28,29^. $H_\text{P}=(123.3 \pm 1.8)\,$ kOe for the $x=0.35$ film and $H_\text{P}=(79.1 \pm 1.8)\,$ kOe for the $x=0.71$ film, indicating that superconductivity is rather limited by orbital effects induced by the externally applied field.

We obtain the Ginzburg–Landau (GL) coherence length $\xi$ through the analysis of $H_{c2}=\phi _{0}/(2\pi {\xi }^2)$. ${\phi }_0=h/2e = 2.07 \times 10^{-7}\,$Oe cm$^2$ corresponds to the magnetic flux quantum, *h* to Planck’s constant, and *e* to the electron charge. The resulting $\xi (T)$ are shown in the inset of Fig. 2b for both alloy compositions. Their diverging characteristics for $T\rightarrow T_\text{c}$ satisfies the GL description of conventional superconductors in the dirty limit $\xi =0.855\sqrt{\xi _\text{0}l}/\sqrt{1-T/T_\text{C}}$. *l* denotes the electron mean free path and in the dirty limit $\xi \approx l$. $\xi _\text{0}=\sqrt{\phi _{0}/2\pi H_\text{c2, 0}}$ can be calculated from the WHH analysis, $\xi _\text{0, x=0.35}=(6.30 \pm 0.01)\,$nm and $\xi _\text{0,\,x=0.71}=(6.80\pm 0.01)\,$nm. Fitting $\xi (T)$, as shown in the inset of Fig. 2b, we obtain $l_\text{x=0.35}=(5.80 \pm 0.18)\,$nm and $l_\text{x=0.71}=(5.65 \pm 0.32)\,$nm for the $x=0.35$ and $x=0.71$ film, respectively. The finding $l_\text{x=0.35, 0.71}<\xi (T)$ based on the $\xi (T)$ analysis explicitly demonstrates dirty limit superconductivity in this HEA type. The observation $l_\text{x=0.35}\approx l_\text{x=0.71}$ is consistent with the comparable mixing entropy of both films, i.e., a comparable amount of atomic scale disorder in the samples.

**Figure 3:**
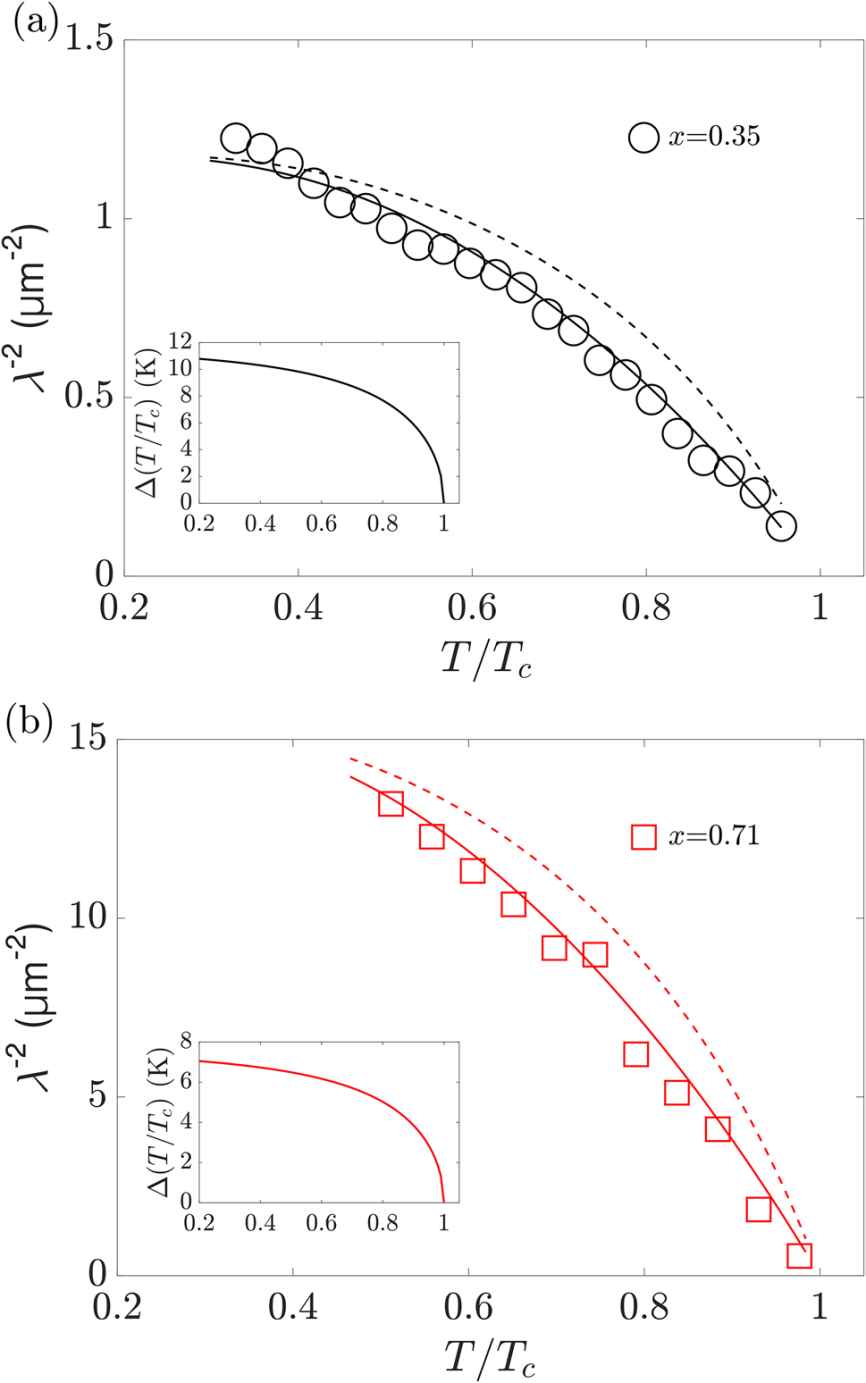
Experimentally determined values of the superconducting penetration depth, $\lambda (T)$, of the (**a**) $x=0.35$ and (**b**) $x=0.71$ films are plotted as $\lambda ^{-2}$ as a function of temperature, *T* (open symbols). The solid (dashed) lines are the corresponding fits to the data using Eq. (1) with $\alpha =1.74$ ($\alpha =2.2$). (**c**) The temperature-dependent quasiparticle gaps $\Delta (T)$ of the $x=0.35$ and $x=0.71$ films obtained from fitting the data in (**a,b**) are shown in the respective insets.

Experimental values of $\xi (T)$ and $H_{c1}(T)$ can be used to determine $\lambda (T)$ by using the relation ${\mu }_0 H_{c1}={\phi }_0/{4\pi \lambda ^2}\ln (\lambda /\xi )$. Knowledge of $\lambda (T)$ can provide valuable insights into the nature of superconductivity in the HEA films. We have quantitatively analyzed $\lambda ^{-2}(T)$ of both HEA films shown in Fig. 3. To this end, we utilize the BCS superfluid density model in the dirty limit^30^,

1 $$\begin{aligned} \rho (T)=\frac{\lambda (0)^{2}}{\lambda (T)^{2}}=\frac{\Delta (T)}{\Delta _\text{0}}\tanh \left[ \frac{\Delta (T)}{2k_\text{B}T}\right] , \end{aligned}$$

where $k_\text{B}$ and $\Delta (T)$ denote Boltzman’s constant and the temperature-dependent superconducting quasiparticle gap, respectively.

Our angle-dependent $H_\text{c1}$ measurements shown in Fig. 1d reveal an isotropic response of the superconducting state to an external magnetic field rotated in the film plane. This observation supports an isotropic superconducting order parameter symmetry, because anisotropic order parameters, such as p- and d-wave, would result in an angle-dependent diamagnetic response. Therefore, we assume an s-wave superconducting order parameter and single-band pairing for our analysis using Eq. (1). The corresponding interpolating BCS gap function reads $\Delta (T)=\Delta _0 \tanh ({\alpha \sqrt{T_\text{C}/T-1}})$ with $\Delta _\text{0}=1.764k_\text{B}T_\text{C}$.

Using this model, we can accurately fit $\lambda ^{-2}(T)$ at both alloy compositions as shown in Fig. 3a. Fitting results in zero temperature penetration depths $\lambda _\text{x=0.35}(0)=(896\pm 4)\,$nm and $\lambda _\text{x=0.71}(0)=(245\pm 2)\,$nm. The calculated quasiparticle gaps, which were used for fitting $\lambda ^{-2}(T)$, are displayed in the corresponding insets. The $\lambda ^{-2}(T)$ of both HEA films with different compositions is in agreement with the weak-coupling BCS limit, $\alpha _\text{x=0.35}=\alpha _\text{x=0.71}=1.74$. It is worth noting that we also observe relatively good agreement between experiment and model at intermediate coupling $\alpha =2.2$ (dashed lines in Fig. 3), which is consistent with previous reports from heat capacity measurements^20^. Overall, our analysis shows that the superconducting state of (TaNb)_1−x_(ZrHfTi)_x_ HEAs can be described with the BCS model for single-band isotropic s-wave superconductivity.

The large degree of compositional disorder is expected to result in a significant on-site potential disorder at the atomic scale^31^. Therefore, the strong dependence of $T_\text{c}$ on *x*, $T_\text{c, x=0.35}=(6.7\pm 1.1)\,$ K and $T_\text{c, x=0.71}=(4.3\pm 1.1)\,$ K, invites speculation on the role of disorder for the $T_\text{c}$ amplitude^21–23^. However, our $H_\text{c2}(T)$ analysis shown in Fig. 2b reveals a comparable mean free path on the order of 5 to 6 nm at both alloy compositions (see Fig. 2b). This observation is consistent with an almost equivalent mixing entropy. It follows that the microscopic disorder is expected to be of comparable strength in both films, despite their rather different elemental composition. Hence, our measurement results cannot support a disorder-driven mechanism as the origin of the observed $T_\text{c}$ variations. It is more likely that the $T_\text{c}$ variations arise from changes to the density of states at the Fermi level induced by electronic doping within a classical BCS framework as reported previously^20^.

## Conclusion

We have experimentally studied the superconducting state of the HEA (TaNb)$_{1-x}$(ZrHfTi)$_{x}$ In thin film form with $x=0.35$ and $x=0.71$ by measuring $H_\text{c1}(T)$ and $H_\text{c2}(T)$. Our analysis of $\lambda (T)$ is in quantitative agreement with the BCS theory of an isotropic single band s-wave superconductor in the weak coupling limit. The analysis of $\xi (T)$ reveals a comparable amount of disorder at both compositions, $l_\text{x=0.35}\approx l_\text{x=0.71}$. Therefore, we can exclude that the observed variations in $T_\text{c}$ originate from a disorder-driven mechanism. Further theoretical and experimental studies will be needed to characterize the low-energy electronic structure at various alloy compositions and its influence on $T_\text{c}$.

Looking ahead, results of such efforts may inform pathways for realizing HEAs with enhanced superconducting $T_\text{c}$. Employing penetration-depth measurements to other superconducting HEAs^8^, such as those crystallizing on the CsCl-type lattice, it will be interesting to test whether weak coupling s-wave superconductivity is a common occurrence in these material systems. While the $T_\text{c}$ variations of bulk superconductivity appear to be independent from disorder, the study of these or other HEAs films in the two-dimensional limit with maximized on-site disorder could offer avenues for exploring $T_\text{c}$ enhancements through multifractal eigenstates near a quantum critical point^22,23,32^.

## Supplementary Information

Supplementary Information.

## Data Availability

The experimental raw data and the corresponding analysis for the reproduction of the presented results are available at 10.5281/zenodo.6673463.

## References

[1] Cantor B, Chang ITH, Knight P, Vincent AJB (2004). Microstructural development in equiatomic multicomponent alloys. Mater. Sci. Eng. A.

[2] Conrad M, Harbrecht B, Weber T, Jung DY, Steurer W (2009). Large, larger, largest-a family of clusterbased tantalum copper aluminides with giant unit cells. II. The cluster structure. Acta Crystallogr. Sect. B.

[3] Gao MC, Yeh J-W, Liaw PK, Zhang Y (2016). A brief review of high entropy alloys and serration behavior and flow units. J. Iron Steel Res. Int..

[4] Guo S, Lu J, Ng C, Liu JCT (2011). Effect of valence electron concentration on stability of fcc or bcc phase in high entropy alloys. Appl. Phys..

[5] Troparevsky MC, Morris JR, Kent PRC, Lupini AR, Stocks GM (2015). Criteria for predicting the formation of single-phase high-entropy alloys. Phys. Rev. X.

[6] Urban K, Feuerbacher JM (2004). Structurally complex alloy phases. Non-Crystallogr. Solids.

[7] Kozelj P, Vrtnik S, Jelen A, Jazbec S, Jaglicic Z, Maiti S, Feuerbacher M, Steurer W, Dolinšek J (2014). Discovery of a superconducting high-entropy alloy. Phys. Rev. Lett..

[8] Sun L, Cava RJ (2019). High-entropy alloy superconductors: Status, opportunities, and challenges. Phys. Rev. Mater..

[9] Gludovatz B, Hohenwarter A, Catoor D, Chang EH, George EP, Ritchie RO (2014). A fracture-resistant high-entropy alloy for cryogenic applications. Science.

[10] Gludovatz B, Hohenwarter A, Thurston KVS, Bei H, Wu Z, George EP, Ritchie RO (2015). Exceptional damage-tolerance of a medium-entropy alloy CrCoNi at cryogenic temperatures. Nat. Commun..

[11] Kou H, Lu J, Li Y (2014). High-strength and high-ductility nanostructured and amorphous metallic materials. Adv. Mater..

[12] Youssef KM, Zaddach AJ, Niu C, Irving DL, Koch CC (2015). A novel low-density, high-hardness, highentropy alloy with close-packed single-phase nanocrystalline structures. Mater. Res. Lett..

[13] Zhang X, Winter N, Witteveen C, Moehl T, Xiao Y, Krogh F, Schilling A, von Rohr FO (2020). Preparation and characterization of high-entropy alloy (TaNb)1x(ZrHfTi)x superconducting films. Phys. Rev. Res..

[14] Harayama Y, Kitagawa J (2021). Superconductivity in Al-Nb-Ti-V-Zr multicomponent alloy. J. Supercond. Novel Magn..

[15] Stolze K, Cevallos FA, Kong T, Cava RJ (2018). Highentropy alloy superconductors on an -Mn lattice. Mater. Chem. C.

[16] von Rohr FO, Cava RJ (2018). High-entropy alloy superconductors on an -Mn lattice. Phys. Rev. Mater..

[17] Vrtnik S, Koželj P, Meden A, Maiti S, Steurer W, Feuerbacher M, Dolinšek J (2017). Superconductivity in thermally annealed Ta-Nb-Hf-Zr-Ti high-entropy alloys. Alloy. Compd..

[18] Xia S, Lousada CM, Mao H, Maier AC, Korzhavyi PA, Sandström R, Wang Y, Zhang Y (2018). Corrigendum: Nonlinear oxidation behavior in pure Ni and Ni-containing entropic alloys. Front. Mater..

[19] Yuan Y, Wu Y, Luo H, Wang Z, Liang X, Yang Z, Wang H, Liu X, Lu Z (2018). Superconducting Ti15Zr15Nb35Ta35 high-entropy alloy with intermediate electron–phonon coupling. Front. Mater..

[20] Von Rohr F, Winiarski MJ, Tao JK, Tomasz C, Joseph R (2016). Effect of electron count and chemical complexity in the Ta-Nb-Hf-Zr-Ti high-entropy alloy superconductor. Proc. Natl. Acad. Sci..

[21] Ma M, Lee PA (1985). Localized superconductors. Phys. Rev. B.

[22] Burmistrov IS, Gornyi IV, Mirlin AD (2012). Enhancement of the critical temperature of superconductors by Anderson localization. Phys. Rev. Lett..

[23] Burmistrov IS, Gornyi IV, Mirlin AD (2021). Multifractally-enhanced superconductivity in thin films. Ann. Phys..

[24] Wencka M (2022). Electronic transport properties of the Al$$_{0.5}$$TiZrPdCuNi alloy in the high-entropy alloy and metallic glass forms. Sci. Rep..

[25] Werthamer NR, Helfand E, Hohenberg PC (1966). Temperature and purity dependence of the superconducting critical field, Hc2. III. Electron spin and spin orbit effects. Phys. Rev..

[26] Pramanik AK, Abdel-Hafiez M, Aswartham S, Wolter AUB, Wurmehl S, Kataev V, Büchner B (2011). Multigap superconductivity in single crystals of Ba0.65 Na0.35 Fe2 As2: A calorimetric investigation. Phys. Rev. B.

[27] Braunisch W, Knauf N, Kataev V, Neuhausen S, Grütz A, Kock A, Roden B, Khomskii D, Wohlleben D (1992). Paramagnetic Meissner effect in bi high-temperature superconductors. Phys. Rev. Lett..

[28] Chandrasekhar BS (1962). A note on the maximum critical field of high-field superconductors. Appl. Phys. Lett..

[29] Clogston AM (1962). Upper limit for the critical field in hard superconductors. Phys. Rev. Lett..

[30] Tinkham M (2004). Introduction to Superconductivity.

[31] Berthold Jäck FZ (2021). Visualizing the multifractal wave functions of a disordered two-dimensional electron gas. Phys. Rev. Res..

[32] Evers F, Mirlin AD (2008). Anderson transitions. Rev. Mod. Phys..

