# Supplementary material for: Evidence for isotropic s-wave superconductivity in high-entropy alloys
Source: Sci Rep. 2022 Jul 27;12:12773. doi: 10.1038/s41598-022-16355-4 (PMC9329343; doi:10.1038/s41598-022-16355-4)
Supplement: Supplementary file 1 — Supplementary Information. [file 41598_2022_16355_MOESM1_ESM.pdf]

Supplementary Information: Evidence for Isotropic s-Wave Superconductivity in High-Entropy Alloys

Casey K.W. Leung,[1] Xiaofu Zhang,[2,3] Fabian von Rohr,[4] Rolf Lortz,[1,5] and Berthold Jäck[1,5]

[1]*Department of Physics, The Hong Kong University of Science and Technology, Clear Water Bay, Kowloon, Hong Kong SAR*
[2]*State Key Laboratory of Functional Materials for Informatics, Shanghai Institute of Microsystem and Information Technology, Chinese Academy of Sciences (CAS), Shanghai 200050, China*
[3]*CAS Center for Excellence in Superconducting Electronics, Shanghai 200050, China*
[4]*Department of Chemistry, Universiät Zürich, Winterthurerstrasse 190, 8057 Zurich, Switzerland*
[5]*IAS Center for Quantum Technologies, The Hong Kong University of Science and Technology, Clear Water Bay, Kowloon, Hong Kong SAR*

(Dated: June 21, 2022)

Appendix A: Structural and chemical properties of the $(TaNb)_{1-x}(ZrHfTi)_x$ thin films

1. Calculation of binary enthalpies

The binary mixing enthalpies of Ta, Nb, Zr, Hf, and Ti are summarized in Table I. The binary mixing enthalpies of Ta-Nb, Zr-Hf, Zr-Ti, and Hf-Ti are all zero that is they neither attract nor repel each other, but mix ideally. The binary mixing enthalpies of other two-element configurations show small positive values that would favor segregation upon long-term annealing. However, the high-entropy alloy (HEA) films investigated in this study are fabricated by using magnetron sputtered at ambient temperatures without post-annealing. Because of the the high mixing entropy, random elemental mixing into a crystalline single phase is favored over possible multi-phase segregation. Consistent with our previous work [1], this understanding is corroborated by our energy-dispersive X-ray spectroscopy and X-ray diffraction measurements presented in the following sub-section. Results from these measurements show a complete elemental mixing and a single phase crystallizing on the body-centered cubic lattice.

TABLE I: Binary mixing enthalpies (in kJ/mol) for unlike atom pairs constituting the $(TaNb)_{1-x}(ZrHfTi)_x$ alloy.

Ta	0	3	3	1
0	Nb	4	4	2
3	4	Zr	0	0
3	4	0	Hf	0
1	2	0	0	Ti

2. Structural and chemical characterizations

The structural and chemical properties of the $(TaNb)_{1-x}(ZrHfTi)_x$ alloy were characterized by using x-ray diffraction (XRD) and energy-dispersive x-ray spectroscopy (EDX) with the scanning electron microscope (SEM), respectively.

Using EDX measurements we have characterized the precise stoichiometric composition of the nominal $x = 0.40$ and $x = 0.75$ films. The relative concentrations of the various elements are listed in Table II. The resulting compositions $x = 0.35$ and $x = 0.71$ closely match the nominal composition targeted during sputter deposition. Spatial EDX mapping conducted on both films, furthermore, reveals a uniform spatial distribution of the five constituent elements of the $(TaNb)_{1-x}(ZrHfTi)_x$ alloy (see Fig. A.1(a) and (b)). This finding reflects the random elemental mixing at the atomic level characteristic for HEAs and is consistent with previous reports [1, 2].

We have conducted 2-Θ XRD scans of the $x = 0.35$ and $x = 0.71$ samples. Fig. A.2 shows the resulting XRD spectrum for measurements on the $x = 0.71$ film using a Cu K(alpha) X-ray source. The diffraction peaks can be labelled with the indices of a body-centered cubic lattice for films at both alloy compositions. The observation of a bcc lattice structure is consistent with previous reports on films and bulk crystals of this material [1, 2].

TABLE II: SEM-EDX analysis of the relative atomic concentrations of the constituent chemical elements of the nominal $x = 0.40$ and $x = 0.75$ films.

Element	Atomic % for $x = 0.40$ film (±0.01%)	Atomic % for $x = 0.75$ film (±0.01%)
Ti	10.37	23.33
Zr	10.32	21.60
Nb	31.99	14.28
Hf	14.49	26.74
Ta	32.82	14.04

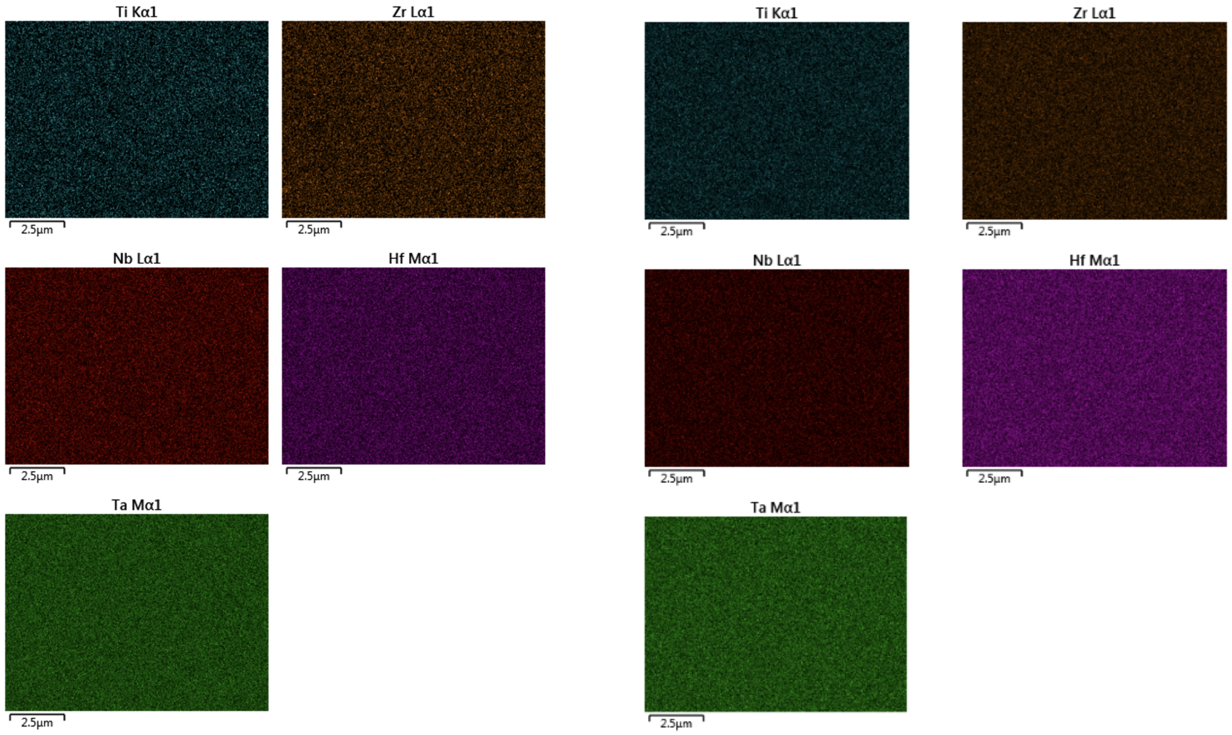

(a) (b)

FIG. A.1: (a) and (b) show spatial maps of the relative atomic concentration for the various constituent elements of the $x = 0.35$ and $x = 0.71$ films, respectively.

Appendix B: Measurements of the magnetic susceptibility of the $x = 0.71$ film

We present the measured magnetic field dependence of $-4\pi M$ measured at various temperatures for the $x = 0.71$ film in Fig. B.1. The magnetic field has been applied in the sample plane along two orientations rotated by 45 °. We have analyzed $-4\pi M(H,T)$ as described in the main text and the resulting H_{c1} values are shown in Fig. 1(d). In Fig. B.2, we show measurements of $-4\pi M$ for a larger range of the external magnetic field applied perpendicular to the sample plane at various representative temperatures. Deviations of $-4\pi M(H)$ from a linear background can be used for determining H_{c2} as described in the main text. The resulting $H_{c2}(T)$ is shown in Fig. 2(b) of the main text.

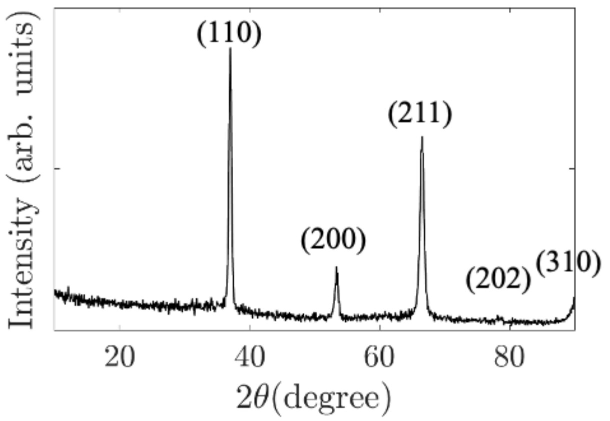

FIG. A.2: X-ray diffraction spectrum of a 2-Θ scan of the $x = 0.71$ film.

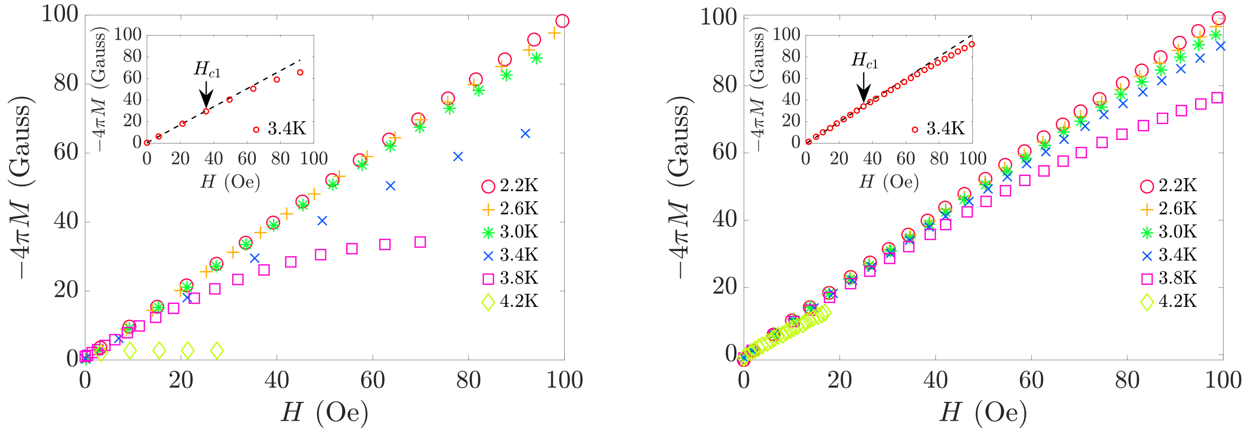

(a)

(b)

FIG. B.1: Measurements of $-4\pi M(H)$ at various indicated temperatures for the $x = 0.71$ film for extracting H_{c1} with the magnetic field H applied along two angles 0° (a) and 45° (b) within the sample plane.

Appendix C: Sample volume for calculation of volume magnetization M

The samples are cut with dimension $a \times b \times c = 2.15 \pm 0.01 \times 2.90 \pm 0.01 \times 0.0011 \pm 0.0001\,\text{mm}^3$ and $2.61 \pm 0.01 \times 2.23 \pm 0.01 \times 0.0010 \pm 0.0001\,\text{mm}^3$ for $x = 0.35$ and $x = 0.71$, respectively. The volume magnetization $-4\pi M$ can be calculated from the magnetic susceptibility χ using $-4\pi M = 4\pi\chi/V$ with $V = a \times b \times c$ as the sample volume.

Appendix D: Determination of the lower critical field H_{c1}

H_{c1} can be determined by analysing the magnetic field H dependence of $-4\pi M$. Upon the application of an external magnetic field $0 < H < H_{c1}$, the superconductor displays a diamagnetic response, which is characterised by a linear $-4\pi M(H)$. Once vortices start to enter the superconducting bulk at $H = H_{c1}$, $-4\pi M(H)$ becomes non-linear for increasing field strengths $H > H_{c1}$.

A linear least-square fit to the diamagnetic response at $H < H_{c1}$ can be used to accurately determine H_{c1} given adequate experimental data quality. Analysing the resulting Pearson coefficient R^2 as shown in Fig. D.1, we can extract H_{c1} as the field at which $R^2 < 1$.

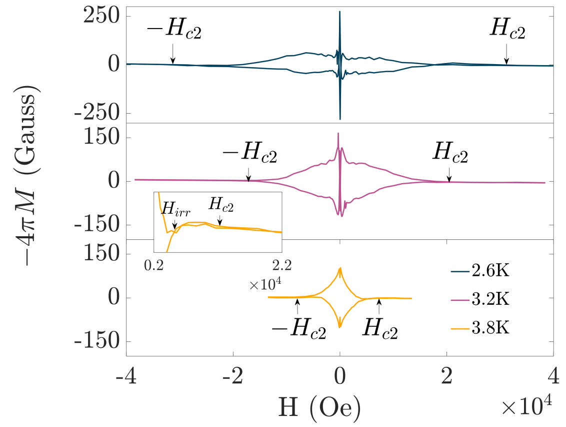

FIG. B.2: Measurements of $-4\pi M(H)$ at representative temperatures of the $x = 0.71$ film over a larger field range for determining H_{c2}, as shown in the inset. H_{irr} denotes the irreversibility field.

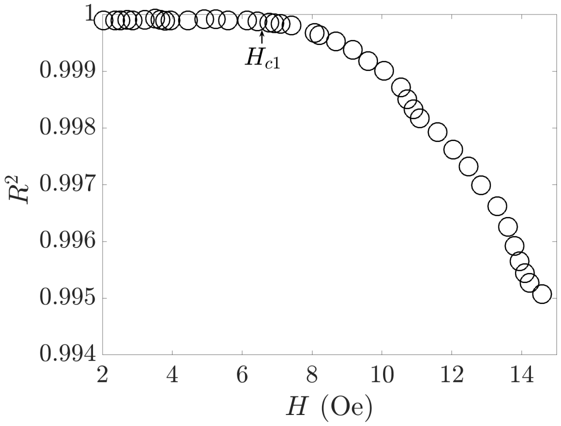

FIG. D.1: The Pearson correlation RR of a linear fit to $-4\pi M(H)$ is plotted. H_{c1} is determined as the field H at which $RR < 1$. An example for $x = 0.35$ and $T = 4\,K$ is shown.

Appendix E: Complete list of determined physical parameters through the analysis of $M(H,T)$

All material parameters that were extracted via the analysis of $-4\pi M(H,T)$ are shown in Table III and IV for the $x = 0.35$ and $x = 0.71$ film, respectively.

TABLE III: Physical parameters of the $x = 0.35$ film. T denotes the temperature, H_{c1} the lower critical field, H_{c2} the upper critical field, ξ the Ginzburg-Landau coherence length, λ the penetration depth, and κ the Ginzburg-Landau parameter.

T(K)	H_{c1}(Oe) (±0.3)	H_{c2}(Oe) (±1600)	ξ(nm) (±0.2)	λ(nm)	$\kappa = \lambda/\xi$
2.2	9.8	67900	7.0	903.32	129.60
2.4	9.6	65100	7.1	913.63	128.32
2.6	9.2	62300	7.3	929.99	127.92
2.8	8.8	60600	7.4	953.24	128.30
3	8.4	57800	7.6	977.55	128.46
3.2	8.2	54600	7.8	985.85	126.23
3.4	7.8	52200	8.0	1013.24	126.81
3.6	7.4	48500	8.3	1038.34	125.25
3.8	7.2	45500	8.6	1044.8	122.06
4	6.9	43500	9.1	1064.21	116.56
4.2	6.7	40100	9.5	1083.07	114.01
4.4	6.3	37100	9.9	1106.69	111.56
4.6	5.8	33500	10.4	1161.17	111.65
4.8	5.4	29800	11.1	1198.56	108.17
5	4.7	26800	11.8	1277.66	108.64
5.2	4.4	23800	12.8	1319.05	103.29
5.4	3.9	20800	13.9	1405.63	101.34
5.6	3.1	18500	15.1	1559.53	103.21
5.8	2.5	14400	17.3	1728.42	100.20
6	2.3	11700	19.8	1805.33	91.09
6.2	1.8	7000	25.6	2028.80	79.31
6.4	1.1	5000	37.5	2548.68	68.04

TABLE IV: Physical parameters of the $x = 0.71$ film. T denotes the temperature, H_{c1} the lower critical field, H_{c2} the upper critical field, ξ the Ginzburg-Landau coherence length, λ the penetration depth, and κ the Ginzburg-Landau parameter.

T(K)	H_{c1}(Oe)(0°) (±3)	H_{c1}(Oe)(45°) (±3)	H_{c2}(Oe)	ξ(nm)	λ(nm)	$\kappa = \lambda/\xi$
2.2	76	74	46500 (±6000)	8.42	275.5	32.72
2.4	70	62	41600 (±2000)	8.90	285.2	32.05
2.6	65	62	38500 (±2000)	9.63	295.2	30.65
2.8	59	58	33100 (±5000)	9.98	310.3	31.09
3	52	50	28503 (±5000)	10.48	331.8	31.66
3.2	50	42	24538 (±3000)	12.50	329.3	26.34
3.4	35	35	21126 (±3000)	14.49	391.7	27.03
3.6	29	27	15658 (±3000)	16.85	430.5	25.55
3.8	23	18	11606 (±200)	19.56	481.0	24.59
4	11	10	6375 (±700)	24.47	725.8	29.66
4.2	3	3	2596 (±300)	35.60	1346.5	37.82

[1] X. Zhang, N. Winter, C. Witteveen, T. Moehl, Y. Xiao, F. Krogh, A. Schilling, and F. von Rohr, Phys. Rev. Res. **2**, 013375 (2020).

[2] V. Rohr, Fabian, Winiarski, M. J, Tao, Jing, Klimczuk, Tomasz, Cava, and R. Joseph, Proceedings of the National Academy of Sciences **113**, E7144 (2016).
